# Curved narratives: cultural representation of spinal deformity in children’s animated films and its psychosocial implications

**DOI:** 10.1007/s43390-025-01156-2

**Published:** 2025-08-06

**Authors:** Rodrigo Muscogliati, Aya Hassanieh, Reine El Ballani, Zeina Najem, Leen Najem, Joe Frem, Khaled Younes, Dima Ezzeddine, Caren Safi, Alexa Chedid, Reem Al Najjar, Elie Najjar

**Affiliations:** 1 Centre for Spinal Studies and Surgery, Queen’s Medical Centre, Nottingham University Hospitals NHS Trust, Nottingham, UK; 2 Hull York Medical School, University of York, York, UK; 3 Gilbert and Rose-Marie Chagoury School of Medicine, Lebanese American University, Byblos, Lebanon; 4 Inria, Paris, France; 5 École Doctorale d’Informatique, Télécommunications et Électronique (ÉDITE), Sorbonne Université, Paris, France

**Keywords:** Spinal deformity, Adolescents, Media representation, Scoliosis, Psychosocial impact

## Abstract

**Purpose:**

Spinal deformities, particularly adolescent idiopathic scoliosis (AIS), are associated with impaired self-image. This study aimed to systematically evaluate how spinal deformities are portrayed in Disney and Pixar animated films and to assess whether recurring visual and narrative stereotypes reflect psychosocial challenges reported by adolescents with AIS.

**Methods:**

A systematic content analysis was conducted of all full-length Disney and Pixar films released from 1989 to 2025. Characters were included if they exhibited consistent anatomical features suggestive of kyphosis, scoliosis, or lordosis. Each character was assessed for physical, social, and moral traits using a standardized checklist. Clinical plausibility was confirmed by an FRCS-trained spine surgeon. Descriptive statistics were used to analyze prevalence and trait distribution.

**Results:**

Forty-eight characters met inclusion criteria, most showing kyphosis (79%). The majority were secondary (42%) or peripheral (35%) figures. Common portrayals included clumsiness (60%), frailty (42%), and frightening demeanor (33%). Only 27% were heroes and 19% were leaders. Female characters were underrepresented (27%).

**Conclusions:**

Spinal deformity is frequently depicted in children’s animation through lenses of physical and social inferiority. These portrayals may contribute to internalized stigma and identity challenges in adolescents with AIS. Clinicians should consider these cultural narratives when discussing appearance-related treatment options.

**Supplementary Information:**

The online version contains supplementary material available at 10.1007/s43390-025-01156-2.

## Introduction

Spinal deformities transcend their biomechanical origins, deeply embedding themselves in psychosocial experiences, particularly during adolescence; a period marked by heightened sensitivity to self-image and peer perception [1, 2]. Adolescents with scoliosis or kyphosis often navigate not only physical challenges but also emotional and social obstacles shaped by how others see them, and by how they come to see themselves. This concept can be better understood through Erikson’s psychosocial development theory [3], particularly the fifth stage, identity versus role confusion, where adolescents strive to form a coherent self-concept and personal identity. During this critical period, external influences such as media play a substantial role in shaping one’s understanding of normalcy and self-worth, especially when compounded by visible physical differences [3]. This paper examines how spinal deformities—especially those portrayed in children’s animated films—may reinforce stigma and affect psychosocial well-being in adolescents with spinal conditions such as AIS. In particular, we explore how these visual and narrative stereotypes may be internalized by young viewers with AIS, shaping their self-image, social confidence, and emotional response to their diagnosis and treatment.

Adolescent Idiopathic Scoliosis (AIS), which affects approximately 0.47–5.2% of adolescents worldwide [4, 5], is associated with elevated levels of anxiety, depressive symptoms, and self-image concerns [6, 7]. These effects are especially pronounced in patients undergoing conservative treatment, such as bracing, which has been linked to discomfort in social settings [8].

Children’s media plays a powerful and often under examined role in shaping these perceptions. As one of the earliest and most pervasive sources of cultural messaging, animated films contribute to how children internalize ideas of normalcy, attractiveness, and deviance [9, 10]. In particular, visible physical differences are frequently exaggerated for narrative effect, often signaling villainy, foolishness, clumsiness, and subservience [11]. When deformity is equated with danger, weakness, or comedic incompetence, young viewers—especially those with similar traits—may learn to associate their own bodies with inferiority or marginality. This association is better expressed using Bandura and Walter’s [12] Social Learning Theory framework, which posits that children and adolescents learn social behaviors and internalize norms through observation, particularly from media. When children are repeatedly exposed to portrayals of physical deformity associated with weakness, villainy, or incompetence, they may adopt these stigmatizing views, including about themselves. This learned association becomes especially impactful during adolescence, a developmental stage Erikson [3] identifies as critical for forming identity and self-concept. Over time, these internalized perceptions can contribute to feelings of inferiority and social marginalization. This phenomenon aligns with critical disability studies, particularly Dolmage’s [13] critique of ableism in cultural narratives, where non-normative bodies are consistently portrayed as deficient or problematic.

Stigma, defined as the social devaluation of individuals based on perceived difference, operates both externally through discrimination and internally through shame and identity threat [14]. Ableism, the structural preference for normate bodies, further reinforces these hierarchies [15]. Yet, despite the global prevalence of spinal deformity, no studies to date have systematically examined how these conditions are portrayed in children’s animation. This represents a significant gap in the literature, especially given the known psychological vulnerability of adolescents with AIS [7].

Understanding how spinal deformity is depicted in popular media is therefore critical. Such representations likely shape patient expectations, influence peer attitudes, and contribute to internalized stigma [16, 17]. For clinicians involved in AIS care, including spine surgeons, psychologists, and rehabilitation teams, recognizing these media-driven narratives is essential for delivering empathic, culturally informed, and patient-centered care.

## Materials and methods

### Film selection

All theatrical, full-length, fully animated films produced or co-produced by Disney or Pixar from 1989 through 2025 were included. The 1989 start date aligns with the release of The Little Mermaid, which marked the beginning of the “Disney Renaissance” a period of highly influential animation with expressive anatomical stylization and global cultural reach [11, 18]. Pixar films were included from their debut in 1995 (*Toy Story*). The end date of 2025 reflects the most current film releases available at the time of data collection. These studios were selected due to their global reach, narrative consistency, and cultural impact in shaping youth perceptions [19, 20].

Exclusion criteria included:

- Short films
- Direct-to-video sequels
- Partially animated or live-action hybrids
- Non-narrative or documentary animations.

Film titles were identified through official studio records and IMDb listings. A complete list of films is available in Online Appendix 1.

### Character inclusion criteria

Characters were included if they displayed at least two of the following observable criteria associated with spinal deformity, as defined by a priori checklist developed by the lead authors, as shown in Table 1.

**Table 1:** Checklist for identifying characters with potential spinal deformity

Criterion	Definition	Visual indicators	Notes
Thoracic kyphosis	Exaggerated convex curvature of the upper spine (posterior thoracic region)	Rounded upper back, “humpbacked” or slouched profile, shoulders hunched forward	Must be visible across ≥ 2 distinct scenes
Lateral deviation (scoliosis/kyphoscoliosis)	Visible curvature of the spine in the coronal plane	Uneven shoulders or hips, “S” or “C”-shaped spinal contour, trunk shift	Should appear consistent and structural, not situational
Hyperlordosis	Exaggerated inward curvature of the lumbar spine	Swayback stance, prominent buttocks or abdomen, exaggerated pelvic tilt	Often appears with exaggerated walking gait or hip sway
Stooped or hunched posture	Chronic forward-flexed posture across multiple scenes	Head and shoulders thrust forward, visible spinal curve even when standing still	Transient hunching (e.g., sneaking) excluded
Structural asymmetry/spinal impairment cues	Features suggesting long-term musculoskeletal imbalance or spinal compromise	Rib hump, shoulder height discrepancy, off-balance gait, narrative descriptors like “crooked” or “twisted”	Also includes use of canes or props implying spinal issues

Characters with temporary postural changes (e.g., due to injury, disguise, or comic effect) were excluded unless those features were persistent and narratively significant.

### Reviewer selection, training, and screening protocol

Nine senior medical students participated as film reviewers, consisting of seven females (authors AH, REB, ZN, LN, DE, CS, and AC) and two males (authors JF and KY). The group was divided into three subgroups of three reviewers each, with each group independently analyzing 22 films. All reviewers underwent a standardized training session covering the spinal deformity checklist and character coding rubric. Coders also took part in a training session focused on identifying spinal deformities—including kyphosis, scoliosis, and lordosis—as well as distinguishing structural deformities from transient or stylistic postures. The training included anatomical review, coding simulations, and a pilot test using three representative films was used to refine definitions and improve inter-rater consistency.

Reviewers independently screened their assigned films and documented characters who met inclusion criteria using a structured data collection form, which was adapted from the study by Liu et al. [11]. Each character’s information was recorded as follows:

- Movie title
- Character name
- Species (human or non-human)
- Verbal status (verbal or non-verbal)
- Gender (male, female, unclear)
- Narrative role (primary, secondary, peripheral)
- Moral alignment (hero, villain, neutral)
- Type of spinal deformity (kyphosis, scoliosis, lordosis)
- Presence of other deformities or health conditions
- Portrayal as scary or frightening
- Clumsiness or movement limitations
- Perceived frailty or reduced capability
- Athleticism
- Intellectual portrayal (intelligent, stupid, neutral)
- Social role (leader, follower, neither).

Subjective narrative traits, such as “frightening demeanor,” “frailty,” and “clumsiness”, were defined in advance using a standardized coding rubric, refined during the reviewer training process and based on prior media studies [11]. A character was classified as “frightening” only if they exhibited at least two of the following features: menacing or distorted facial expressions (e.g., scowls, hollow eyes, and sharp teeth), dark or ominous visual styling (e.g., cloaks, skeletal frames, and shadows), threatening dialog or tone, or hostile behavior repeated across multiple scenes. Similar criteria were applied to other traits, with clear visual or behavioral indicators outlined for each.

Each film was independently reviewed by three trained coders. A character’s traits were only recorded if at least two reviewers independently agreed. Disagreements or borderline cases were resolved through group discussion to reach consensus. While anatomical plausibility of spinal deformity was reviewed by a consultant spine surgeon, narrative and emotional traits were resolved entirely within the trained reviewer panel, following the rubric criteria.

### Clinical validation and inter-rater agreement

All included characters were independently reviewed for anatomical plausibility by a consultant spine surgeon (FRCS-trained). Discrepancies were resolved via panel discussions moderated by a senior researcher with experience in qualitative analysis. This process yielded significant reviewer consensus, with only 4 characters being removed due to reviewer disagreements, yielding a total of 48 characters to be included in the study.

### Bias mitigation strategies

In line with criteria for trustworthiness in qualitative research; credibility, dependability, confirmability, and transferability [21–23], the following safeguards were implemented:

- Reviewers were **blinded to the study’s hypothesis** and instructed to adhere strictly to the deformity checklist and coding rubric.
- Reviewers were oriented to review the movies independently.
- Trait assessments (e.g., moral alignment, intelligence) were completed independently of physical analysis.
- All data were collected **before** any analysis to avoid post hoc rationalization.
- Inclusion decisions were **reviewed by clinical experts** to prevent overdiagnosis or misinterpretation of stylized features.

### Data management and analysis

All data were anonymized and compiled into a Microsoft Excel spreadsheet for analysis, designed to ensure standardized formatting and facilitate cross-checking. Descriptive statistics were used to quantify the prevalence and traits of characters with spinal deformities. Traits such as character gender were selected based on the characters voice, appearance, or pronouns used throughout the movies.

In addition, descriptive grouping of narrative patterns was used to summarize recurring character archetypes, informed by existing disability tropes in media literature [11], where three dominant themes emerged:

- *The Frail Elder* Depictions of age-associated deformity linked with vulnerability
- *The Monstrous Outsider* Use of spinal deformity to signal villainy or otherness
- *The Comic Sidekick* Characters with spinal traits framed as humorous or bumbling.

### Ethical considerations

As this study involved publicly available media and no human participants, formal ethical approval was not required. All coding and analyses were conducted using de-identified media content in accordance with ethical standards for media studies.

## Results

### Character selection

**Fig. 1:**
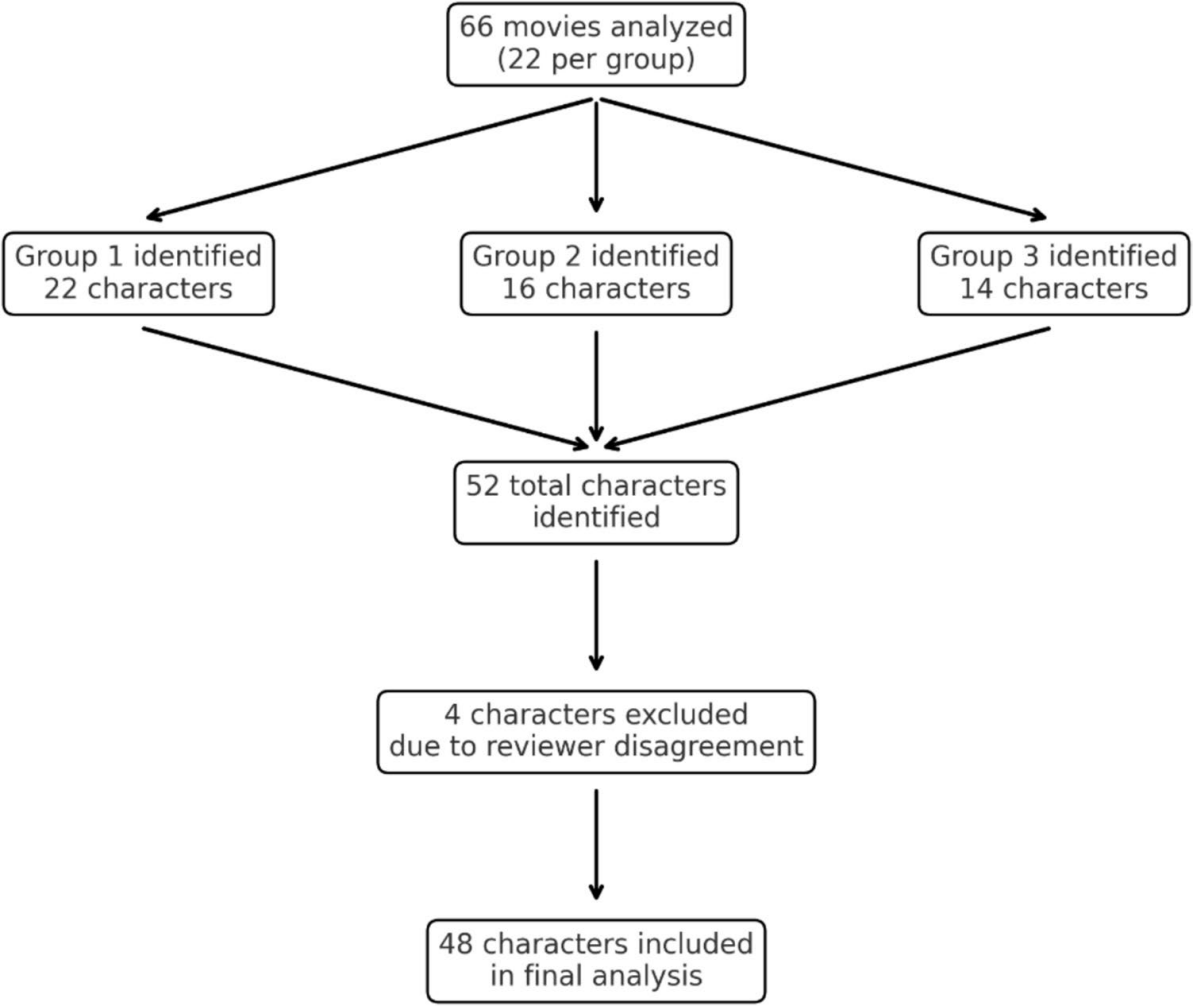
Visual summary of the movie selection and character identification process, illustrating how the final sample of 48 characters was derived

A total of 66 films were analyzed, of which 36 (54.5%) included characters with spinal deformities. From these 36 films, 48 unique characters were identified, as depicted in Fig. 1. The following results examine these 48 animated characters, featured in Disney and Pixar productions released between 1989 and 2025, through both visual and narrative analysis.

1. Prevalence and types of spinal deformities
  Kyphosis dominated portrayals (79%), followed by scoliosis/kyphoscoliosis (19%) and lordosis (2%), as shown in Table 2 and Fig. 2.

**Table 2:** Distribution of spinal deformity types

Deformity type	Count	Percentage
Kyphosis	38	79%
Scoliosis/kyphoscoliosis	9	19%
Lordosis	1	2%

**Fig. 2:**
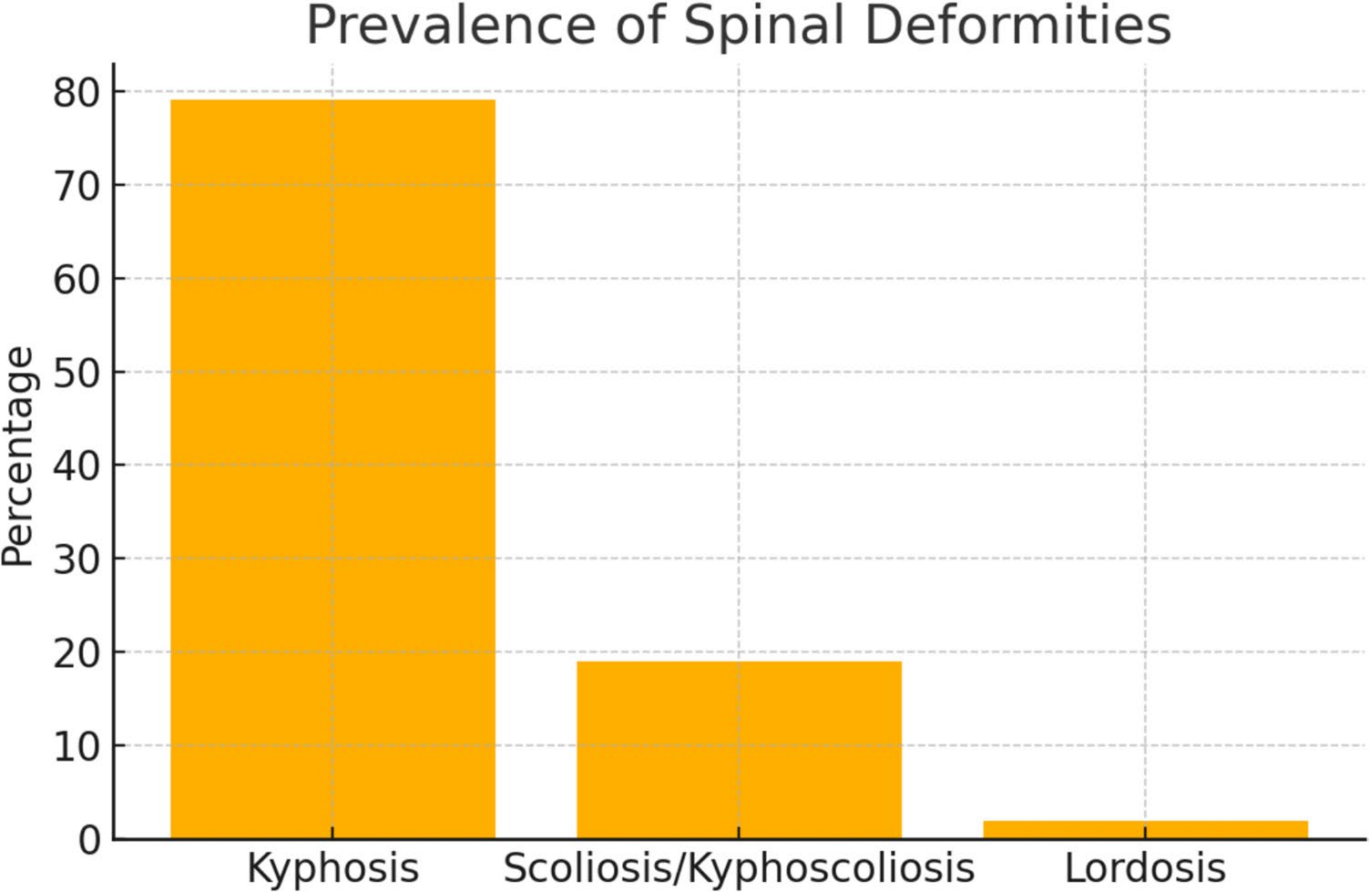
Distribution of spinal deformity types: Bar chart illustrating the prevalence of spinal deformities among animated characters, highlighting kyphosis as the most common
2. Narrative roles and moral alignment
  Characters were primarily secondary (42%) or peripheral (35%), with few as protagonists (21%). Moral alignment skewed neutral (54%) or villainous (19%), as shown in Table 3, Figs. 3 and 4.

**Table 3:** Narrative role and moral alignment of characters

Role	Count	Percentage	Moral alignment	Count	Percentage
Primary	10	21%	Hero	13	27%
Secondary	20	42%	Villain	9	19%
Peripheral	17	35%	Neutral/neither	26	54%

**Fig. 3:**
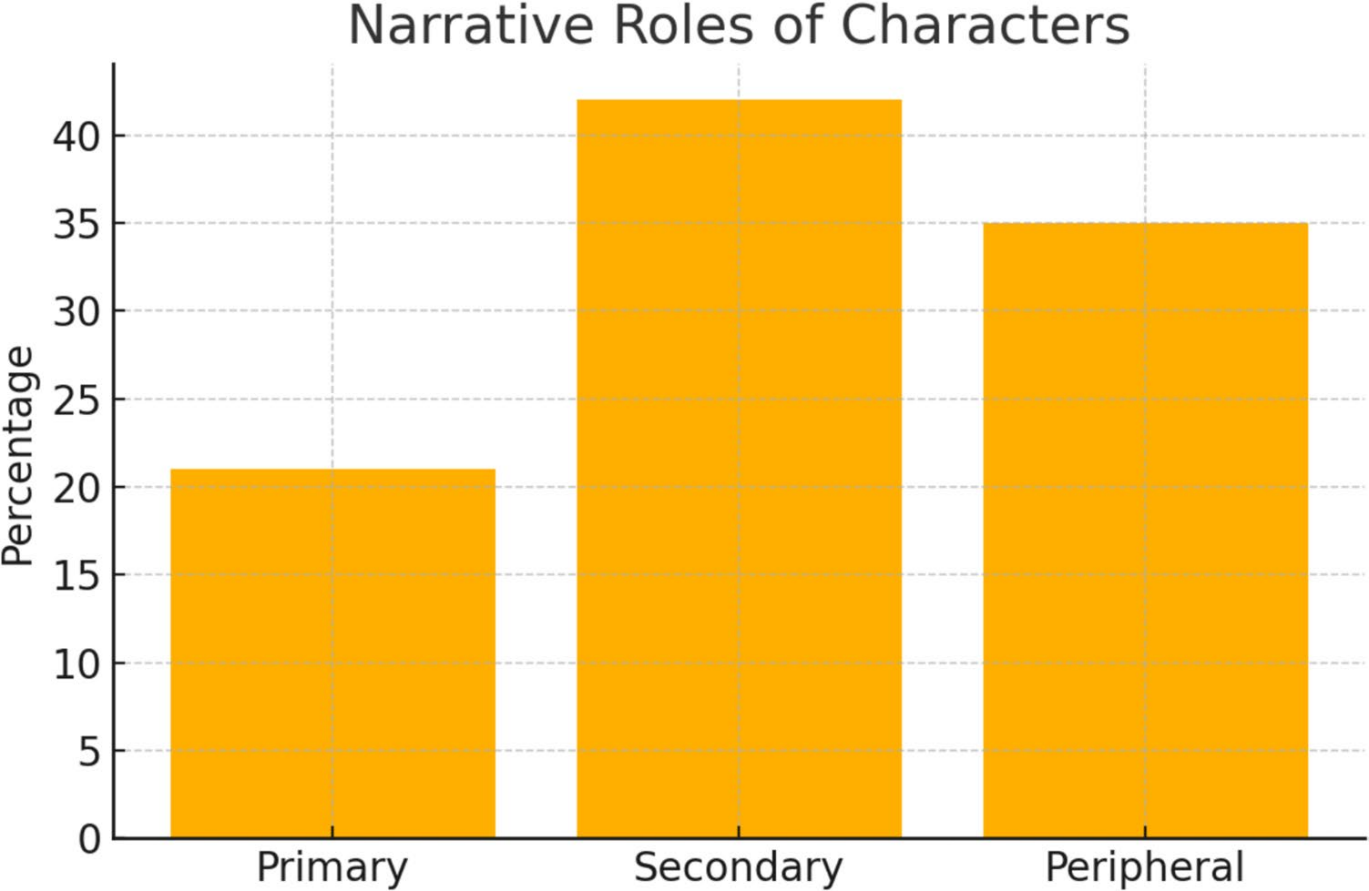
Narrative roles of characters: Bar chart showing the distribution of characters’ narrative roles, with a majority in secondary or peripheral positions

**Fig. 4:**
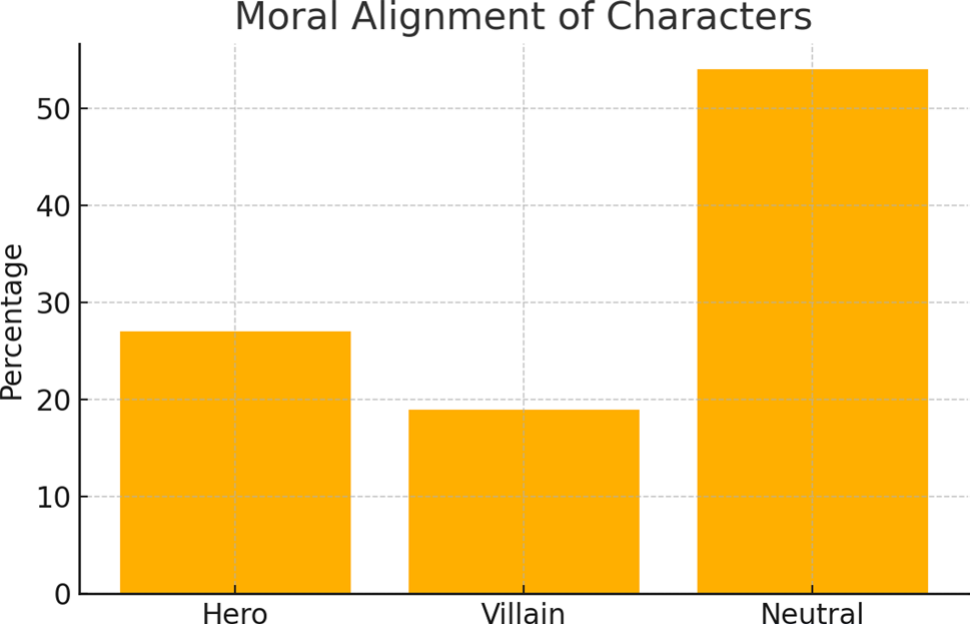
Moral alignment of characters: Bar chart depicting the moral alignment of characters, indicating a predominance of neutral portrayals
3. Behavioral and physical stereotypes
  Over half (60%) were clumsy/slow-moving, while 33% were framed as frightening. Frailty (42%) and non-athleticism (77%) were common, as shown in Table 4 and Fig. 5.

**Table 4:** Behavioral and physical portrayals

Trait	Yes	No	% Yes
Scary/frightening	16	32	33%
Clumsy/slow-moving	29	19	60%
Frail/less capable	20	28	42%
Athletic	11	37	23%

**Fig. 5:**
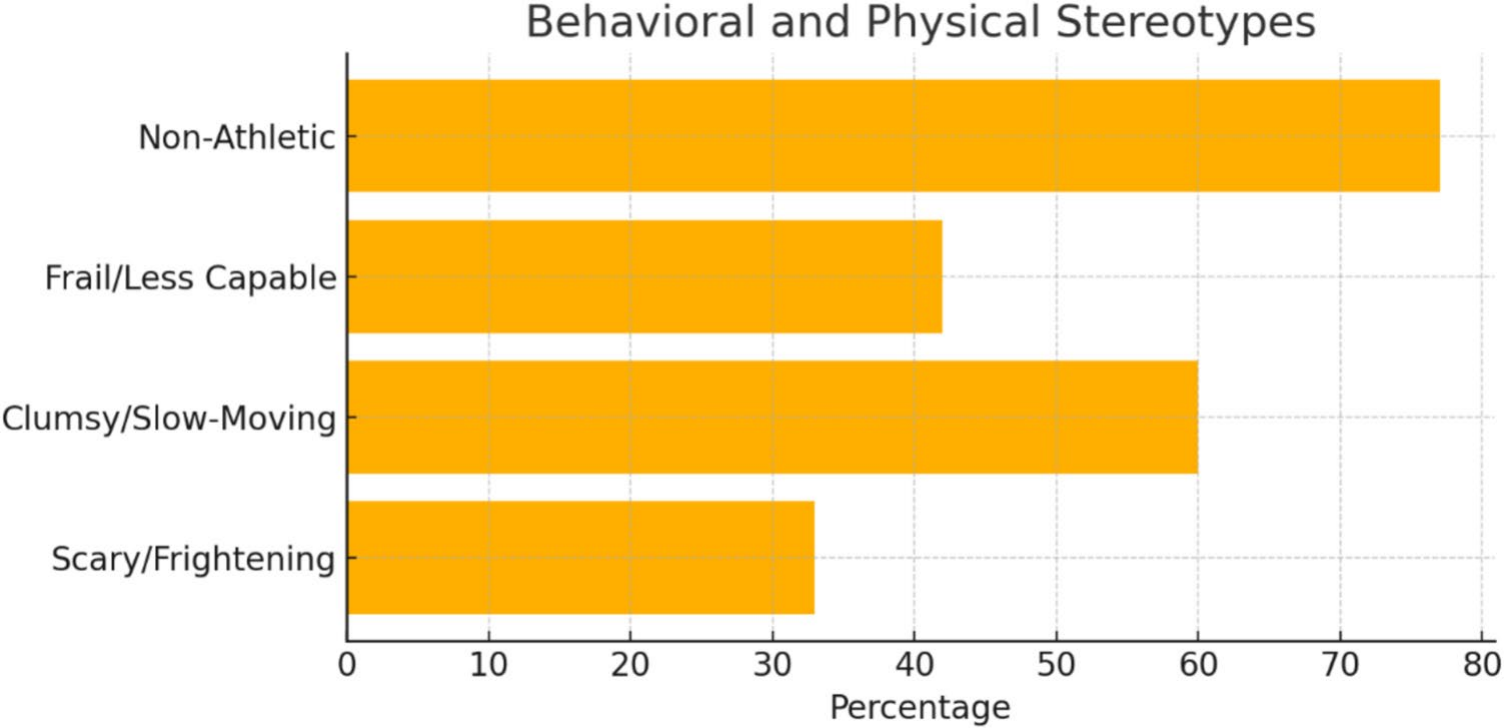
Behavioral and physical stereotypes: Horizontal bar chart presenting the frequency of stereotypical traits, such as clumsiness and frailty, among characters
4. Intellectual and social framing
  Few characters were leaders (19%) or explicitly intelligent (42%), shown in Table 5, Figs. 6 and 7.

**Table 5:** Cognitive and social roles

Intelligence	Count	Percentage	Social role	Count	Percentage
Intelligent/wise	20	42%	Leader	9	19%
Neutral/unclear	25	52%	Follower	18	38%
Stupid/foolish	3	6%	Neither	21	44%

**Fig. 6:**
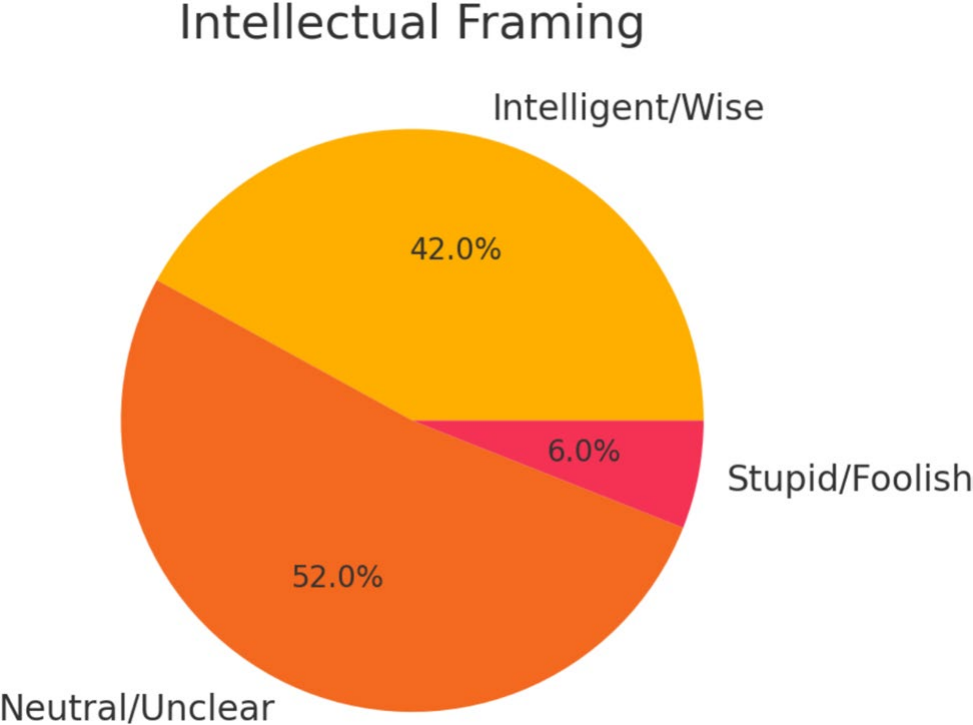
Intellectual characterization of characters: Pie chart illustrating the distribution of characters’ intellectual portrayals, with a significant portion labeled as neutral or unclear

**Fig. 7:**
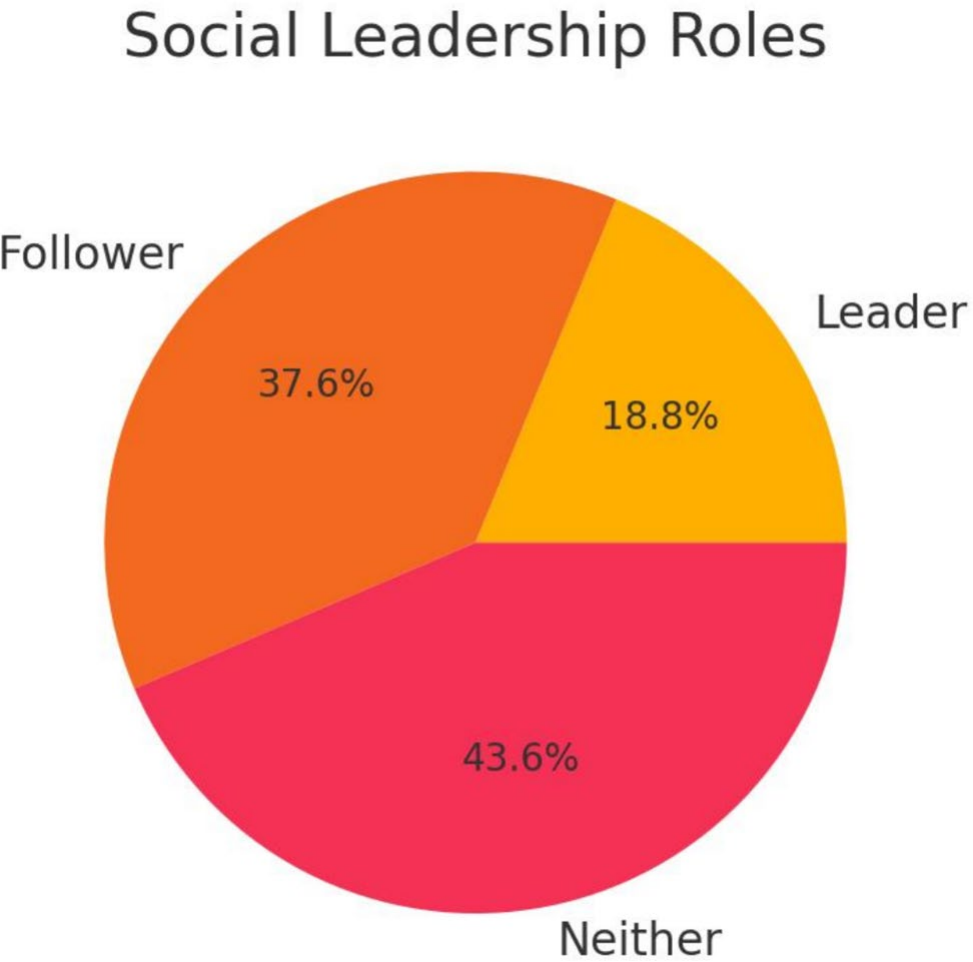
Social roles in terms of leadership and followership: Pie chart showing the social roles of characters, emphasizing the limited depiction of leadership positions
5. Gender representation
  Gender representation within the sample revealed a marked imbalance. Of the 48 characters identified, 35 were male (73%) and 13 were female (27%). No characters were coded as having unclear or non-binary gender identity.
6. Human vs non-human representation
  In terms of species, 28 characters (58%) were human and 20 (42%) were non-human. Non-human portrayals included animals, mythical beings, and anthropomorphic figures.
  While the prevalence of kyphosis was similar across both groups, their narrative framing differed. Non-human characters were more frequently portrayed with humorous or bumbling traits, while human characters were more often associated with frailty or a frightening aesthetic.

### Key patterns

1. **Kyphosis as the dominant visual trope **(79%).
2. **Marginal narrative roles**: 77% secondary/peripheral.
3. **Negative stereotyping**: 60% clumsy, 33% frightening, and 42% frail.
4. **Limited agency**: 19% leaders, 38% followers.

## Discussion

This study provides the first systematic analysis of how spinal deformities are portrayed in mainstream animated films, with a focus on works produced by Disney and Pixar from 1989 to 2025. Among the 48 characters identified as exhibiting structural spinal traits consistent with kyphosis, scoliosis, or lordosis, the majority were depicted as peripheral, physically frail, socially subordinate, or morally ambiguous. Athleticism, leadership, and confidence were notably scarce in this group, while clumsiness and frightening attributes were common. These patterns mirror a broader media tradition in which physical difference is used as a narrative shorthand for otherness, incompetence, or deviance [11], echoing Hall’s [24] concept of “representational regimes” that shape public understanding through repetition and visual coding.

While these portrayals may not be intentionally stigmatizing, they reflect entrenched representational patterns that unconsciously reproduce ableist narratives. Even in the absence of overt hostility, such visual scripting contributes to symbolic exclusion, whereby adolescents with AIS come to see their bodies as out of place, both on screen and in society.

The consistent use of kyphosis as a visual cue for weakness or villainy reinforces ableist ideologies that equate physical deviation from the norm with moral or social deficiency. Suggests what Pescosolido and Martin [25] term the “stigma complex”: a system of interrelated social meanings and structures through which difference is marked, moralized, and enacted. Within this framework, spinal deformity constitutes a visibly discrediting attribute that invites labeling, stereotyping, social separation, and symbolic exclusion. In this context, spinal deformity becomes not just a medical condition but a symbolic marker of diminished value and otherness [13].

### Psychosocial interpretation and relevance to AIS

These portrayals are especially relevant in the context of Adolescent Idiopathic Scoliosis (AIS), a condition that typically emerges during early adolescence—a critical period for identity formation and social comparison [2, 3]. Research consistently shows that adolescents with AIS experience elevated psychological distress, including anxiety, diminished self-esteem, and impaired quality of life [7, 8]. These effects are not merely cosmetic concerns; studies have shown that subjective perceptions of deformity, rather than curve severity, are more predictive of mental health outcomes and satisfaction with care [4, 26, 27].

Although the majority of characters in our study were portrayed with kyphosis rather than scoliosis, the cultural messaging embedded in these portrayals remains highly relevant to AIS. Research suggests that adolescents are more affected by the perceived visibility of their condition than its precise structural classification, and that visual cues of “abnormal posture”—whether kyphotic or scoliotic—can evoke similar psychosocial responses [4, 6, 7, 26]. The consistent use of curved spines as shorthand for frailty, comedic incompetence, or villainy may therefore reinforce stigma and self-consciousness among AIS patients. These portrayals contribute to both public stigma—the broader societal devaluation of physical difference—and internalized stigma, in which individuals absorb cultural messages and apply them to their own bodies. This internalization has real-world implications for how adolescents view their condition, respond to clinical advice, and interpret their need for bracing or surgery.

Our findings also suggest differences in how deformity is narratively framed between human and non-human characters. Non-human figures, such as animals or mythical creatures, were more commonly used for comic relief and often appeared as eccentric or awkward sidekicks. Human characters, on the other hand, were more frequently associated with frailty, isolation, or villainous roles. Despite tonal variation, deformity in both groups was linked to marginalization, reinforcing its role as a visual marker of social otherness.

These depictions may also shape how adolescents with AIS interpret themselves in relation to social norms. As many of these characters are visually coded as ‘other’—through curved postures, awkward movement, or marginal roles—young viewers with spinal deformity may internalize those associations. Even if they recognize the difference between their condition and the exaggerated kyphosis shown on screen, they may nonetheless feel implicated in the same social scripts of weakness, ridicule, or exclusion. This perceived mirroring may heighten self-consciousness during a formative period, particularly if the child has not yet disclosed their condition or begun treatment. As a result, animated portrayals may indirectly influence identity formation, body image, and willingness to engage in care.

Furthermore, cultural representations may also influence enacted stigma, the real-world discrimination, and exclusion faced by adolescents with scoliosis. Although studies on peer bullying and spinal deformity are limited, there is strong precedent in stigma literature, showing that negative cultural portrayals contribute to teasing, social marginalization, and even educational disadvantage in children with other visible or labeled differences [25].

### Clinical implications

For clinicians managing AIS, particularly in appearance-driven treatment scenarios, these findings underscore the importance of understanding patients’ cultural and psychological context. Appearance-related concerns are now widely recognized as major drivers of surgical decision-making, sometimes surpassing physical function as the key motivator for operative intervention [26–28]. In this context, patient-reported outcomes, such as the SRS-22 self-image and satisfaction domains [29], should be interpreted not only as reflections of physical impairment but also as indicators of psychosocial burden shaped by long-term exposure to cultural scripts. Adolescents may come into clinic already shaped by media messages that equate spinal curvature with weakness or unattractiveness, and this framing can influence how they evaluate bracing compliance, surgical necessity, or expectations of “normalcy” postoperatively.

Clinicians have an opportunity and arguably an ethical obligation to counteract stigmatizing narratives. This includes validating concerns about appearance, addressing internalized stigma directly, and helping patients reframe their experiences through empowerment and positive identity construction. Drawing from the FINIS proposed by Pescosolido and Martin [25], such interventions should occur at multiple levels: individual, interpersonal, clinical, and cultural.

### Public health and education implications

Beyond the clinical setting, these findings carry significant implications for public health communication, disability advocacy, and educational practice. Children’s animated films continue to serve as early and potent sources of social learning, shaping norms around bodies, difference, and value [12]. As our analysis demonstrates, these narratives often rely on narrow and stigmatizing portrayals of physical deformity, particularly spinal differences.

Collaborations between pediatric clinicians, disability scholars, and content creators could promote more inclusive storytelling practices. Such efforts might include consulting on character design, participating in disability representation advisory boards, or advocating for narrative audits that assess how physical difference is depicted. Models like GRALE [30, 31] and initiatives by organizations such as RespectAbility offer emerging frameworks for ethically engaging with media producers to reduce ableist bias.

In educational contexts, integrating media literacy into patient programming or school health curricula could help adolescents critically evaluate cultural messages about body norms. Media literacy interventions, particularly those focused on body image, have shown promising results in building resilience and decreasing internalized stigma [32, 33]. Encouraging young people with AIS to reflect on and challenge these messages may strengthen their capacity for self-advocacy, a key protective factor in navigating chronic conditions and disability identity development [15, 25].

### Limitations and future research

While this study employed a standardized visual and narrative rubric, the interpretation of stylized animation is inherently subjective. To mitigate this, anatomical assessments were confirmed by a spine surgeon, and inter-rater agreement among trained coders was high. Nonetheless, future research could expand this analysis to other studios, genres, or media forms, such as television, social media, or video games. Additionally, qualitative studies with adolescents who have spinal deformities, using methods such as focus groups, media diaries, or visual elicitation, could provide deeper insight into how these portrayals are received and internalized. Such research would enrich our understanding of lived stigma and resilience, particularly when framed through models like the Stigma Complex [25] or Social Learning Theory [12]. Furthermore, the movie reviewers were senior medical students, whose perceptions and identification of spinal deformities may differ from those of other viewer populations, such as children and adolescents who are the primary consumers of these films. The majority of film reviewers were female, reflecting the composition of the voluntary research team at the time. While training and rubric-based standardization aimed to reduce bias, we acknowledge that this demographic imbalance may have influenced interpretive framing.

## Conclusion

Spinal deformity in mainstream animation is rarely portrayed neutrally. Instead, it is often employed as a visual shorthand for frailty, incompetence, or villainy, reinforcing damaging cultural narratives about physical difference. These portrayals are not merely symbolic; they contribute to the psychosocial environment in which adolescents with scoliosis navigate identity, self-worth, and medical decision-making. For clinicians, researchers, and media creators alike, acknowledging and addressing these cultural messages are critical to advancing disability equity, both in health and in representation.

## Supplementary Information

Below is the link to the electronic supplementary material.

Supplementary file1 (XLSX 16 KB)

## Data Availability

All data were derived from publicly available media sources. No new data were generated.
